# Synopses of Editorial Board

**DOI:** 10.1080/13577149877876

**Published:** 1998-12


					Sarcoma (1998) 2, 129 132
SYNOPSES OF EDITORIAL BOARD
David M. Panicek, M.D.
David M. Panicek, M.D. is a graduate of Cornell University and Cornell University Medical College.
He is currently Associate Professor of Radiology at Cornell University Medical College and Director of
Educational Programs in Radiology at Memorial Sloan Kettering Cancer Center. Dr Panicek is a
member of the Commission on Research and Technology Assessment of the American College of
Radiology, and is a member of the Roster of Distinguished Scienti(R) c Advisors for the RSNA Research
and Education Fund. He is also a member of the American Joint Committee on Cancer (AJCC) Task
Force on Soft Tissue Sarcoma. Dr Panicek is a manuscript reviewer for Radiology, American Journal of
Roentgenology, Journal of Thoracic Imaging, Journal of Computer Assisted Tomography, and Sarcoma, and
serves on the Editorial Board of Sarcoma. His primary research activities include the imaging of primary
and recurrent musculoskeletal neoplasms; MR imaging assessment of musculoskeletal sarcoma response
to preoperative therapy; and ef(R) cacy studies in radiology. Dr Panicek was the lead investigator of the
Radiology Diagnostic Oncology Group (RDOG) multi-institutional study of CT and MR imaging in the
local staging of musculoskeletal neoplasms, funded by the National Cancer Institute.
1357-714 X/98/030129 04 $9.00 O 1998 Carfax Publishing Ltd
130 Synopses
Dr C. L. Harmer
Dr Clive Harmer quali(R) ed at the Westminster Hospital London in 1963 and undertook House jobs
there followed by Senior House Of(R) ce and Registrar posts in the Radiotherapy Department. In 1967
he moved to the Royal Marsden Hospital to become Senior Registrar and three years later went to
work with Dr Henry Kaplan on Hodgkin's Disease at Stanford University California as Damon
Runyon Fellow. He was appointed Consultant Radiotherapist at St Luke's Hospital Guildford in 1970
which was a joint appointment with the Royal Marsden Hospital at Sutton. Three years later he
became Radiotherapist to St George's Hospital and established the Combined Sarcoma Clinic at the
Royal Marsden together with Dr Eve Wiltshaw. Over the past decade he has successively been Vice
Chairman, Chairman, Chief of Radiotherapy Services, and Head of Radiotherapy, recently relinquish-
ing this post to become Head of the Thyroid Unit. He was previously both Vice Chairman and
Chairman of the Medical Staff Committee of the Royal Marsden Hospital. He has published widely on
the treatment of soft tissue sarcoma. Chapters include Sarcoma of the Head and Neck, The Manage-
ment of Sarcoma in the Adolescent, and Conformal Plus Three-Dimensional Radiotherapy Planning.
Synopses 131
Robert J. Grimer
Robert Grimer is a Consultant Orthopaedic Oncologist working at the Royal Orthopaedic Hospital
Oncology Service in Birmingham, UK. This Unit is one of the largest tertiary referral centres for primary
bone and soft tissue sarcomas in the United Kingdom. He has extensive experience of limb salvage
surgery, using endoprosthetic replacements for bone tumours and has a particular expertise in limb
salvage procedures for children using extensible and debral CC's. He serves on the MRC Bone and Soft
Tissue Sarcoma Panels and is a member of the European Musculo-Skeletal Oncology Society and the
International Society of Limb Salvage.
132 Synopses
Lee J. Helman, M.D.
Dr Helman received his M.D. from the University of Maryland School of Medicine in 1980. He
completed his Clinical Training in Internal Medicine at Barnes Hospital, Washington University in
1983, and in Medical Oncology at the National Cancer Institute Pediatric Oncology Branch and
Medicine Branch in 1986. He became the head of the Molecular Oncology Section of the Pediatric
Oncology Branch in 1993, and Chief of the Pediatric Oncology Branch in 1997. Dr Helman is a
member of the American Association for Cancer Research, American Society of Clinical Oncology,
American Society of Clinical Investigation, and is a member of the Board of Directors of the
Connective Tissue Oncology Society.

				

